# Increased Risk of Colorectal Cancer in Patients With Celiac Disease: A Population-Based Study

**DOI:** 10.7759/cureus.36964

**Published:** 2023-03-31

**Authors:** Somtochukwu Onwuzo, Antoine Boustany, Mustafa Saleh, Riya Gupta, Chidera Onwuzo, Jessy Mascarenhas Monteiro, Favour Lawrence, Chinenye Emeshiobi, Juliana Odu, Imad Asaad

**Affiliations:** 1 Internal Medicine, Cleveland Clinic Foundation, Cleveland, USA; 2 Faculty of Medical Sciences, Lebanese University, Beirut, LBN; 3 Faculty of Medicine, Kasturba Medical College, Mangalore, IND; 4 Internal Medicine, General Hospital Lagos Island, Lagos, NGA; 5 Internal Medicine, Ross University School of Medicine, Bridgetown, BRB; 6 Internal Medicine, Mercy Hospital Fort Smith, Fort Smith, USA; 7 Public Health, The University of Toledo, Toledo, USA

**Keywords:** database, population-based study, cancer screening, colorectal cancer, celiac disease

## Abstract

Background and aim: The association between celiac disease (CD) and the development of small bowel lymphoproliferative disorders and esophageal adenocarcinoma has been established in the literature. However, there is only a little evidence demonstrating an increased risk of colorectal cancer (CRC) in patients with CD. Hence, we conducted a cross-sectional population-based study to evaluate the risk of developing CRC in patients who have had a diagnosis of CD.

Methodology: We used a commercial database (Explorys Inc, Cleveland, OH), which includes electronic health records from 26 major integrated US healthcare systems. Patients aged 18-65 years were included. Patients with inflammatory bowel disease (IBD) were excluded. Multivariate analysis using backward stepwise logistic regression was performed to calculate the risk of developing CRC in potential confounders. A two-sided P-value <0.05 was considered statistically significant.

Results: 79,843,332 individuals were screened in the database and 47,400,960 were selected in the final analysis after accounting for inclusion and exclusion criteria. Using a stepwise multivariate regression analysis, the odds of having CRC among patients with CD was 10.18 (95% CI 9.72-10.65) (P-value <0.001). The odds also remained high among males 1.49 (95% CI 1.36-1.63), African Americans 1.51 (95% CI 1.35-1.68), patients who have type 2 diabetes mellitus (T2DM) 2.71 (95% CI 2.66-2.76), are smokers 2.49 (95% CI 2.44-2.54), are obese 2.21 (95% CI 2.17-2.25), and are alcoholic 1.72 (95% CI 1.66-1.78).

Conclusion: Our study demonstrates that patients with CD are frequently found to have CRC even when adjusting for common risk factors. This adds to the literature and helps spread awareness to clinicians that the effects of CD are not only limited to the small bowel as the disease tends to involve other parts of the gastrointestinal tract also, especially the colon. The threshold to screen patients with CD should be considered to be lowered.

## Introduction

Celiac disease (CD), sometimes called gluten-sensitive enteropathy, is an autoimmune-mediated inflammatory disease affecting the small intestine. It is caused by dietary gluten and other related protein sensitivity in genetically predisposed individuals. The global prevalence of CD is approximately 1.4% with positive serological samples and 0.7% with biopsy [1]. Despite being a disease with a high global burden, it is severely underdiagnosed, and hence no proper treatment is given until the later course of the disease [2]. The diagnosis of CD is based on positive serological antibodies and biopsy in susceptible individuals [3]. Comprehensive investigations show a strong association between CD and the development of small bowel lymphoproliferative disorders and esophageal adenocarcinoma [4]. 

Few studies have demonstrated a possible increased risk of colorectal cancer (CRC) in patients with CD. However, other studies have shown no risk or slight risk in the development of CRC. According to research by Lasa et al., there is an increased risk of developing CRC in patients with CD [5]. However, according to a study by Volta et al., an increase in CRC was only seen in individuals above the age of 60 [6]. Other studies showed no increase in the prevalence of colonic neoplasia in patients with CD [7-11].

Multiple hypothetical theories can explain this controversial association. Most patients in the studies mentioned above were on a strict gluten-free diet (GFD), which may have reduced the disease burden, which leads to a decrease in the incidence of CRC. There may be a decrease in the occurrence of epithelial malignancies in CD with an increase in intestinal intraepithelial lymphocytes. Additionally, because untreated CD impairs the absorption of fat or fat-soluble substances, such as hydrocarbons and probable co-carcinogens, which are linked to the development of CRC, it has been proposed that untreated CD may have a preventive effect against the disease [12]. On the other hand, the possibility that CD may contribute to colonic neoplasia is believed to stem from exaggerated T-cell response inflammatory response, hence promoting tumorigenesis. 

However, due to the limited evidence available, more studies are necessary to determine whether there is an actual association. Hence, we sought to evaluate the risk of developing CRC in patients diagnosed with CD.

## Materials and methods

Methodology

Data from our cohort were collected using Explorys, a verified, multicenter, and continuously updated database created by IBM Watson Health. With a total of around 360 hospitals and more than 70 million patients nationwide, Explorys is made up of the electronic health data of 26 separate healthcare systems. Explorys uses SNOMED-CT, or Systematized Nomenclature of Medicine-Clinical Terms, to describe disorders. The diagnosis is made by the individual healthcare practitioners, who subsequently input the data in the form of SNOMED-CT codes into the database. According to the clinical component being examined, the database pools enormous amounts of deidentified data from both inpatient and outpatient settings that may be formed into a variety of cohorts. Individual patient information, including test or imaging results, is not kept on file by Explorys. Several organizations, and consequently healthcare providers, may use different methods to diagnose various medical diseases because the data are compiled from numerous organizations. The database is heavily dependent on individual organizations giving accurate data because of the way it is set up, which makes it impossible to analyze the manner of diagnoses. Explorys is a platform that complies with the Health Insurance Portability and Accountability Act (HIPAA), hence the Institutional Review Board's permission is not necessary. The use of this database has been approved in a number of specialties, including gastroenterology, hematology, and cardiology [13,14].

Patient selection

A cohort of patients with a SNOMED-CT diagnosis of “Celiac disease” and “Colorectal Cancer” between 1999 and May 2022 was identified. Patients aged 18-65 years were included. We excluded individuals who have had a diagnosis of inflammatory bowel disease.

Covariates

We treated male gender, African American ethnicity, and Hispanic race as variables. Confounding factors associated with CRC were also identified and collected if SNOMED-CT diagnoses were available. These were type 2 diabetes mellitus (T2DM), smoking, obesity, and alcoholism.

Statistical analysis

To account for confounding from the covariates listed above, we conducted 256 searches to explore every probability. A univariate analysis was conducted initially for all the variables. A multivariate analysis using backward stepwise logistic regression was performed to calculate the risk of developing CRC. A conservative significance threshold of 0.01 was used to determine the qualification of data entry into or deletion from the model. African American and Hispanic ethnicities, male patients, individuals with CD, type 2 diabetics, smokers, obese patients, and alcohol users were included in the logistic regression. A two-sided P-value <0.05 was considered as statistically significant, and all statistical analyses were performed using R version 4.0.2 (R Foundation for Statistical Computing, Vienna, Austria, 2008). 

## Results

Among the 47,400,960 individuals screened in this database, there were a total of 82,880 subjects with CRC. There were more males with CRC than females. Interestingly, while the majority of subjects were Caucasians, 13% were African Americans. About one-third of the patients with CRC had underlying hyperlipidemia while a quarter of patients had BMI ≥30 (Table 1).

**Table 1:** Baseline characteristics of patients with colorectal cancer and control T2DM: type 2 diabetes mellitus; *H. pylori*: *Helicobacter pylori*; IBS: irritable bowel syndrome.

		CRC (%)	No CRC (%)
	Total	n=82,880	n=47,318,080
Sex	Male	46,840 (56.52)	25,954,180 (54.85)
	Female	36,040 (43.48)	21,363,900 (45.15)
Race	Caucasian	57,950 (69.92)	24,364,530 (51.49)
	African American	10,900 (13.16)	5,390,950 (11.39)
	Hispanic	1,150 (1.39)	744,890 (1.57)
	Asian	2,460 (2.97)	798,890 (1.69)
	Others	10,399 (12.56)	16,021,901 (33.86)
Comorbidities	Type 2 diabetes mellitus	14,390 (17.36)	2,185,810 (4.62)
	Celiac disease	1,890 (2.28)	82,470 (0.17)
	Hyperlipidemia	25,230 (30.44)	4,548,180 (9.61)
	Obesity	18,060 (21.79)	3,468,550 (7.33)
	H. pylori	280 (0.33)	52,980 (0.11)
	IBS	2,810 (3.39)	578,560 (1.22)
Substance abuse	Smoking	14,450 (17.43)	2,777,000 (5.87)
	Cannabis	1,560 (1.88)	485,860 (1.03)
	Alcohol	3,780 (4.56)	818,240 (1.73)

In univariate analysis, the odds of having CRC in subjects with CD were 14.02 (95% CI 13.40-14.65), males 2.19 (95% CI 1.80-2.50), African Americans 2.22 (95% CI 2.10-2.45), patients who have T2DM 4.31 (95% CI 4.23-4.39), are smokers 3.36 (95% CI 3.30-3.42), are obese 3.49 (95% CI 3.43-3.54), and are alcoholic 2.78 (95% CI 2.69-2.87) (Table 2).

**Table 2:** Calculation of the risk of developing CRC using univariate analysis CRC: colorectal cancer; T2DM: type 2 diabetes mellitus.

	CRC	
	OR (95% CI)	P-value
African American	2.22 (2.10-2.45)	<0.001
Celiac disease	14.02 (13.40-14.65)	<0.001
T2DM	4.31 (4.23-4.39)	<0.001
Smoking	3.36 (3.30-3.42)	<0.001
Obesity	3.49 (3.43-3.54)	<0.001
Alcoholism	2.78 (2.69-2.87)	<0.001
Male	2.19 (1.80-2.50)	<0.001

**Figure 1:**
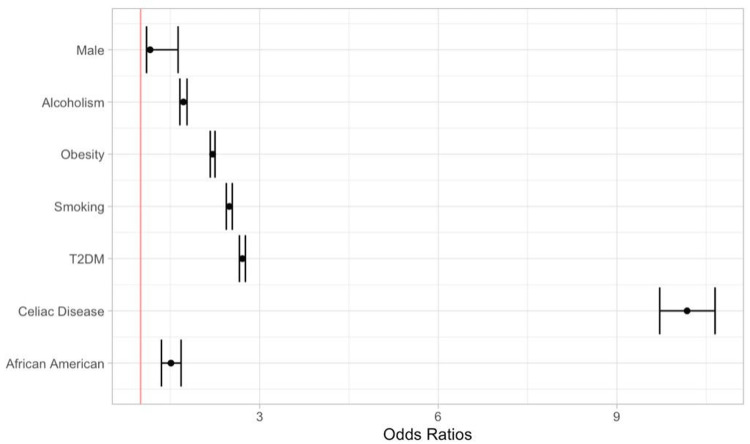
Forest plot for the risk of developing colorectal cancer using multivariate analysis T2DM: type 2 diabetes mellitus.

In multivariate analysis, the odds of having CRC among patients with CD was 10.18 (95% CI 9.72-10.65) while the odds also remained high among males 1.49 (95% CI 1.36-1.63), African Americans 1.51 (95% CI 1.35-1.68), patients who have T2DM 2.71 (95% CI 2.66-2.76), are smokers 2.49 (95% CI 2.44-2.54), obese 2.21 (95% CI 2.17-2.25), and are alcoholic 1.72 (95% CI 1.66-1.78) (Figure 1).

## Discussion

The present study aimed to investigate the association between CD and the incidence of CRC. Results indicated that CD was associated with an increased risk of CRC. Notably, when examining the odds ratios (ORs), CD emerged as a significant risk factor for CRC (OR 10.18) with an even higher risk than other known risk factors such as smoking, obesity, alcohol abuse, and T2DM.

Research conducted to understand the controversial CD and CRC association has been increasing lately. A study by Ilus et al., which examined the incidence of malignancies in 32,439 adult celiac patients from 2002-2011 using standardized incidence ratios (SIRs), found elevated SIRs for non-Hodgkin lymphoma, small intestinal cancer (adenocarcinoma, carcinoid, and stromal tumors), and colon cancer among celiac patients compared to the general population [15]. Han et al. propose that individuals with CD and other organ-specific immune conditions are at a heightened risk for developing cancer, particularly locally invasive [9]. Additionally, the observation of a 6.89 hazard ratio (HR) for small intestine cancer in individuals with CD further supports this increased risk [16]. Furthermore, Poyrazoglu et al. highlighted a new association between CD and extraintestinal malignant neoplasms such as esophageal squamous cell carcinoma [4].

Regarding CRC, the literature is limited but is increasingly starting to examine this association. In a retrospective case-control study of 57 cases and 118 controls, Lasa et al. found a significant association between newly diagnosed CD in adult patients and increased odds of CA (OR 2.95) [5]. In the said study, emphasis was placed on utilizing colonoscopy quality indicators to improve the adenoma detection rate. Additionally, a logical approach to gluten exposure was adopted in the study population. This is in line with previous research by Pereyra et al., which demonstrated that non-compliance to a GFD is associated with a higher risk of colonic adenoma [17]. This finding raises the hypothesis that untreated CD may contribute to an increased risk of CRC. This emphasis should have been placed in previous studies that did not establish a clear association [18].

Goldacre et al., in 2018, conducted a record linkage study involving 14,114 cases and 457,071 controls to investigate the association between Crohn's disease, ulcerative colitis, and CD with CRC. The results of the study indicated an increased risk for CRC in individuals with Crohn's disease (OR 1.27), ulcerative colitis (OR 1.25), and CD (OR 1.16) [19]. The possibility that CD may contribute to neoplasia outside the small bowel through mechanisms related to malabsorption, abnormal inflammation, or the effects of gluten is biologically plausible. Immune mechanisms of cancer and the pathophysiology of immune-mediated diseases are intricately interwoven, with dysfunctional regulatory T cells and TH17 cells promoting inflammation and distant tumorigenesis potentially regulated by microbiota-derived signals [16].

Several studies have produced inconsistent results with the association established in our research. The retrospective analysis of data from the Italian Registry of Complications of CD by Volta et al. revealed that celiac patients on a strict GFD had a dramatically reduced risk of developing colon cancer, with an SIR of 0.07. This result suggests that gluten withdrawal may play a role in preventing the proliferation of normal enterocytes, which is an early step in neoplastic cell transformation [6]. Gluten peptides have been reported to trigger proliferative signaling in a cell line derived from human colon carcinoma, and other mechanisms may be at play [20]. Moreover, it is crucial to consider other factors that may play a role in CRC development, such as very low gluten load reaching the colon, different histology of the colon mucosa, and reduced absorption of fats and carcinogens through the inflamed intestinal mucosa [21].

Two meta-analyses, by Han et al. and Lasa et al., have investigated this relationship and have yielded somewhat contradictory results [9,10]. Han et al. included a total of 17 studies and found that CD is associated with an increased risk of all malignancies, particularly gastrointestinal malignancies. In contrast, Lasa et al., which included three studies, did not find an increased risk of malignancies in patients with CD. The inconsistency in results between these two meta-analyses could be attributed to the number of studies included and the specific characteristics of the studies included. Furthermore, it is essential to consider that other factors such as age at presentation, duration of disease before compliance to GFD, the definition of peri- and post-diagnosis period, criteria for diagnosis of CD, colonoscopy quality indicators used, the retrospective and prospective design employed, and confounding social factors may also influence results and contribute to the heterogeneous pool of studies in the meta-analysis.

The significance of these findings is multifaceted, as they indicate that individuals with CD may be at a heightened risk for colon cancer and perhaps more frequent screening than the average risk population. Given the potential risk identified, it is crucial to prioritize early and accurate diagnosis of CD and adherence to a GFD while concurrently minimizing other factors, such as socioeconomic and lifestyle factors, to lower the overall risk of CRC. Our study forms the basis for future research by providing a large dataset pooled analysis from 360 US hospitals. It is also important to share these results with healthcare professionals and raise awareness among the general public regarding the potential link between CD and cancer.

Given the potential clinical implications of these findings, additional research is needed to fully understand the association between CD and cancer risk and establish any causal relationship; for example, study designs that consider subgroup characteristics and minimize the reliance on routine hospital data. Moreover, to gain a comprehensive understanding of the relationship, it is necessary to examine other potential influencing factors and conduct further studies to uncover the underlying mechanisms of this association.

This study has several limitations. First, it is a retrospective population-based study, which has inherent limitations regarding the conclusions drawn from the data. Important clinical factors such as the timing of diagnosis, compliance with a GFD, and duration of the diagnosis were not taken into account. In addition, the study did not control for a family history of CRC, advanced adenoma, and prior screening colonoscopies, which may have led to biased results. The nature of this study also made it impossible to obtain information on the criteria used for the diagnosis of CD or the quality indicators used in colonoscopies to detect CRC.

## Conclusions

In conclusion, increased colonic mucosal sensitivity to gluten triggers an exaggerated immune response. T lymphocyte-led cellular hyperproliferation and subsequent tumorigenesis is a plausible explanation as to why our study depicts that patients with CD are frequently found to have CRC even when adjusting for common risk factors of CRC. Literature investigating this topic over the years has reached varying results, hence no current guidelines on colonoscopy screening in patients with CD. Nevertheless, further research is needed in this field to ascertain to what degree the age of celiac diagnosis, compliance to a strict GFD, lifetime diagnostic, and/or therapeutic endoscopic evaluation for other health comorbidities play a role in either increasing or decreasing the risk of CRC in patients with CD.
